# Understanding physical activity participation in people living with rheumatoid arthritis

**DOI:** 10.1093/rap/rkaf146

**Published:** 2025-12-23

**Authors:** Julia New-Tolley, Kathryn A Dyer, Susan Lester, Jessica Stanhope, Simon Burnet, Kimberley Ting, Sajini Basnayake, Suman Murthy, Elizabeth Hoon, Susanna Proudman, Catherine L Hill

**Affiliations:** Adelaide Medical School, University of Adelaide, Adelaide, SA, Australia; Rheumatology Department, Queen Elizabeth Hospital, Woodville South, SA, Australia; Rheumatology Department, Queen Elizabeth Hospital, Woodville South, SA, Australia; Rheumatology Department, Queen Elizabeth Hospital, Woodville South, SA, Australia; School of Public Health, University of Adelaide, Adelaide, SA, Australia; Rheumatology Department, Queen Elizabeth Hospital, Woodville South, SA, Australia; Rheumatology SA, Adelaide, SA, Australia; Rheumatology SA, Adelaide, SA, Australia; Rheumatology SA, Adelaide, SA, Australia; Medplus SA, Kent Town, SA, Australia; Adelaide Medical School, University of Adelaide, Adelaide, SA, Australia; School of Public Health, University of Adelaide, Adelaide, SA, Australia; Adelaide Medical School, University of Adelaide, Adelaide, SA, Australia; Rheumatology Department, Royal Adelaide Hospital, Adelaide, SA, Australia; Adelaide Medical School, University of Adelaide, Adelaide, SA, Australia; Rheumatology Department, Queen Elizabeth Hospital, Woodville South, SA, Australia; Rheumatology Department, Royal Adelaide Hospital, Adelaide, SA, Australia

**Keywords:** rheumatoid arthritis, physical activity, exercise, exercise guidelines, muscle strengthening, muscle resistance, barriers, perceptions, health professionals, confidence

## Abstract

**Objective:**

We examined self-reported physical activity (PA) levels [metabolic equivalents expended (METs)/week and muscle strengthening/resistance activities] in Australians with RA and explored related factors.

**Methods:**

A cross-sectional questionnaire assessed whether participants met PA guidelines—defined as >600 METs-min/week of activity and two or more muscle strengthening sessions/week of at least 10 min duration. The questionnaire also collected demographics, RA characteristics including disease activity using the RAPID3 assessment, barriers and support from health professionals. Results were compared between those who did and did not meet the guidelines.

**Results:**

Most participants [120/180 (67%)] expended >600 METs/week but only 29% (53/180) participated in muscle strengthening/resistance activities at least twice a week for >10 min at a time. Less than a quarter [43/180 (24%)] were considered to be meeting PA guidelines (i.e. both the METs and muscle strengthening guidelines) and those who did were less likely to have high disease activity as per the RAPID3 Disease Activity Score (4.7% *vs* 6.6%; *P* = 0.003). A lower proportion of those who met the PA guidelines reported RA symptoms as a barrier compared with those who did not meet the guidelines (42% *vs* 84%; *P* = 0.03) and a higher proportion reported confidence in their ability to exercise safely (51% *vs* 31%; *P* = 0.02). They were also more likely to have received information from health professionals regarding PA that they perceived to be useful (65.1% *vs* 40.2%; *P* = 0.0004). Those who met the PA guidelines were more likely to believe it was possible to meet the recommendations for muscle strengthening (74.4% *vs* 48.9%; *P* = 0.006).

**Conclusion:**

Most people with RA are not meeting full PA guidelines, largely due to low muscle-strengthening activity. Disease activity, confidence and professional support appear to be associated with PA participation.

Key messages

- Less than a third of people with rheumatoid arthritis were doing physical activity that helps strengthen muscles.
- People who met physical activity guidelines had lower disease activity and were less likely to consider rheumatoid arthritis symptoms a barrier.
- People with rheumatoid arthritis who felt confident and informed regarding physical activity were more likely to be meeting physical activity guidelines.

## Introduction

RA, the most common form of inflammatory arthritis, had a global prevalence of 208.8/100 000 in 2020 [1]. Untreated, RA can cause significant pain and disability, systemic involvement and increased mortality, mostly due to increased cardiovascular risk [2].

The impact of RA can be reduced by early, intensive pharmacological and non-pharmacological treatment. Physical activity (PA) improves disease activity and function in people with RA [3, 4], with additional benefits on comorbidities, e.g. cardiovascular risk, RA-related cachexia (loss of muscle mass due to underlying disease) and osteoporosis [5, 6]. Given evidence for efficacy and safety, PA in line with the World Health Organization (WHO) public health recommendations has been recommended by the EULAR as a standard of care [7]. In addition, the Australian Clinical Care Standards for adults with RA recommend access to services that can support participation in PA [8].

The current global recommendations on PA for health released by the WHO recommend adults should do ≥150 min of moderate-intensity aerobic PA or ≥75 min of vigorous-intensity aerobic PA/week, or an equivalent combination of moderate- and vigorous-intensity activity, in sessions of ≥10 min [9]. Additionally, ‘muscle strengthening exercise’ (defined by the WHO as ‘physical activity and exercise that increases skeletal muscle strength, power, endurance and mass, e.g. strength training, resistance training or muscular strength and endurance exercises’) of major muscle groups should be done on ≥2 days/week [9]. National PA guidelines, including in Australia, the UK and the USA, are in line with these recommendations [10–12].

Systematic review evidence shows PA levels are lower in those with RA compared with the general population [13]. Accelerometry data have shown that people with RA are less likely to participate in PA of moderate or higher intensity compared with those without RA [14]. A recent Swedish study found that 41% of people with RA met the minimum recommended time per week for moderate or vigorous PA at two time points over 7 years [15]. A further Swedish study found that while 69% of people with RA met the recommended time per week of health-enhancing PA at the time of the study, only 14% reported muscle strength training [16]. There is no literature regarding PA in Australians with RA.

Barriers and enablers to PA in people with RA include RA-specific disease factors, psychological status (e.g. fear of increased symptoms), environmental factors (e.g. poor accessibility and weather), lack of PA education and support from health providers [17, 18]. Fear of movement (kinesiophobia) is also weakly correlated with lower PA levels [19].

The purpose of this study was to determine PA behaviours of people with RA living in South Australia by describing the amount and type of self-reported PA undertaken compared with the Australian PA guidelines; whether there are differences in demographics, RA characteristics, barriers and support from health professionals between those who meet the guidelines and those who did not; and to gain an understanding of unmet needs regarding PA support.

## Methods

### Design and participants

We conducted a cross-sectional study. People with RA were recruited through advertising with a QR code linking to the survey, from September to December 2022, with fliers in waiting rooms of two public rheumatology outpatient clinics (Queen Elizabeth Hospital and Royal Adelaide Hospital) and two private rheumatology clinics in Adelaide, South Australia. Additionally, rheumatologists working in these clinics reminded patients who had RA of the invitation. An advertisement was also placed in the consumer organisation Arthritis South Australia newsletter.

Eligibility criteria were being ≥18 years of age, living in South Australia and having rheumatologist-diagnosed RA. Participants recruited online were excluded if they were not taking a DMARD, to reduce the risk of including participants without an RA diagnosis.

### Data collection

The questionnaire (11 sections, 41 items; Supplementary Data S1) collected data about demographics, medical conditions, PA, potential barriers and enablers and beliefs regarding exercise and was delivered through REDCap [20, 21]. Items were based on validated questionnaires, where possible, and was piloted with two researchers, one registered nurse, one physiotherapist and three adults with RA. Minor adjustments to wording and formatting were made following piloting. Pilot testing indicated the questionnaire took 15–20 min to complete.

### Physical activity

The International Physical Activity Questionnaire – Short Form (IPAQ-SF) was used to self-report PA [22]. Data were scored to estimate energy expenditure in metabolic equivalents (METs) [23]. The IPAQ-SF is reliable, with good repeatability when categorising those who participate in >600 METs/week (which roughly translates to 150 min of moderate or 75 min of vigorous PA/week). Criterion validity has shown fair to moderate agreement of the IPAQ-SF compared with accelerometry data [22].

We asked about recreational PA using a list of activities from 2020 AusPlay data [23] and arthritis recommendations [24], and participants had the opportunity to add other activities to the list. Participants were asked to report only PAs with sessions lasting ≥10 min and were asked how often they participated in the activity. Activities were classified as potentially muscle strengthening using definitions from the Health Survey for England and AusPlay survey appendix [24, 25].

#### Physical activity outcome

Participants were classified as meeting the Australian PA guidelines [10] if they expended >600 METs/week and engaged in ≥10 min of muscle strengthening PA two or more times per week.

### Other information collected

We compared age, gender, socio-economic status, occupational status, level of education, language spoken at home, health literacy, recruitment, BMI, smoking status, comorbidities, RA disease duration, current medication use, RA disease activity, fatigue, enablers and barriers to PA, kinesiophobia, source of information about physical activity, health professional support and perceptions regarding PA, including the importance to well-being, confidence in participation and achievability of meeting PA guidelines (see Supplementary Table S1 for details), between those who did and did not meet the PA guidelines.

#### Enablers and barriers

The validated Inflammatory Arthritis Facilitators and Barriers to physical activity questionnaire (IFAB) was used to assess known barriers and enablers of PA [17, 26]. This questionnaire comprises 10 items, including disease factors, psychosocial status, social support and environmental factors. Facilitators of PA are scored positively, barriers are scored negatively and a score of 0 indicates no impact on PA. Four items could be considered either barriers or enablers (scored −10–10), three items considered barriers only (scored −10–0) and three items considered enablers only (scored 0–10). The total score is the sum of the items (−70–70), with lower scores indicating more barriers and higher scores indicating more enablers.

#### Understanding unmet needs

Open-ended questions (Box 1) were used to deepen our understanding of barriers and enablers to physical activity and explore areas of unmet need through the voice of people with RA.

Box 1. Open-ended questions

- Is there anything you would like to find out from your rheumatologist regarding exercise?
- Any comments about these (Australian physical activity) guidelines?
- Would anything help you to feel more confident to participate in exercise?
- Is there anything else you would like to tell us about exercise and rheumatoid arthritis?

### Analysis

Statistical analysis was performed in Stata version16 (StataCorp, College Station, TX, USA). Data were tabulated as mean (s.d.), median ([interquartile range (IQR)] or proportions, as appropriate. Participants were categorised into those who met the PA guidelines and those who did not. To compare these two groups the chi-squared (exact) test was used for categorical data, two-sample *t*-test for continuous normally distributed data and Mann–Whitney U test or Jonckheere-Terpstra test for trend (exact) for continuous data that were not normally distributed. A 5% level of significance was used.

#### Qualitative analysis

Responses to the questions in Box 1 were read and re-read from a clinician viewpoint, with the overarching question in mind, ‘How can health professionals help to facilitate PA uptake in RA?’. Codes were generated by one author (J.N.-T.) line by line and compiled manually into overarching themes. The themes were reviewed with an experienced qualitative researcher (E.H.). Authors were blinded to demographic and PA status.

### Permissions

This study complied with the Declaration of Helsinki. Ethics approval was granted by the Central Adelaide Local Health Network Human Research Ethics Committee (reference 16537). Written informed consent from all participants was obtained prior to the questionnaire.

## Results

### Demographics and health status

The questionnaire was commenced by 227 people and 180 (79.3%) completed the survey, which were included in our analyses. The mean age was 62 years and most participants were female (Table 1).

**Table 1:** Participant demographics and health status.

Variable	Total (*N* = 180)	Meeting physical activity guidelines^a^ [*n* = 43 (24%)]	Not meeting physical activity guidelines [*n* = 137 (76%)]	*P*-value^b^
Female, *n* (%)	126 (70.0)	25 (58.1)	101 (73.7)	0.059
Age, years, mean (s.d.)	61.6 (14.0)	62.8 (15.6)	61.2 (13.4)	0.531^c^
Recruitment, *n* (%)				
Public clinic	98 (54.4)	16 (37.2)	82 (59.9)	0.039
Private clinic	55 (30.6)	20 (46.5)	35 (25.6)	
Online	20 (11.1)	5 (11.6)	15 (11.0)	
Other	7 (3.9)	2 (4.7)	5 (3.7)	
Employment, *n* (%)				
Retired	72 (40.0)	22 (51.2)	50 (36.5)	0.110
Full-time	42 (23.3)	12 (27.9)	30 (21.9)	
Part-time	29 (16.1)	4 (9.3)	25 (18.3)	
Other^d^	37 (20.6)	5 (11.6)	32 (23.4)	
Education level, *n* (%)				
Did not finish high school	38 (21.1)	5 (11.6)	33 (24.1)	0.107^e^
Finished high school	31 (17.2)	9 (20.9)	22 (16.1)	
Trade/apprenticeship	14 (7.8)	2 (4.7)	12 (8.8)	
Certificate/diploma	51 (28.3)	13 (30.2)	38 (27.7)	
Bachelor’s degree	46 (25.6)	14 (32.6)	32(23.4)	
First language English	170 (94.0)	43 (100)	127 (93.0)	0.120
Reading difficulty^f^	12 (7.0)	0 (0)	12 (9.0)	0.072
Socio-economic status				
1 (lowest)	35 (19.4)	5 (11.6)	30 (21.9)	0.564^e^
2	39 (21.7)	12 (27.9)	27 (19.7)	
3	38 (21.1)	12 (27.9)	26 (19.0)	
4	39 (21.7)	4 (9.3)	35 (25.6)	
5 (highest)	29 (16.1)	10 (23.3)	19 (13.9)	
BMI^g^, *n* (%)				
Underweight/normal range	47 (26.7)	18 (42.9)	29 (21.6)	0.013^e^
Overweight	48 (27.3)	10 (23.8)	38 (28.4)	
Obese	81 (46.0)	14 (33.3)	67 (50.0)	
Current smoker, *n* (%)	22 (12.2)	1 (2.3)	21 (15.3)	0.030
Rheumatic Disease Comorbidity Index, median (IQR)^h^	1 (0–3)	1 (0–2)	1 (0–3)	0.145^i^
Disease duration (years), *n* (%)				
0-5	48 (26.7)	10 (23.3)	38 (27.7)	0.952^e^
6–10	41 (22.8)	12 (27.9)	29 (21.2)	
>11	91 (50.6)	21 (48.8)	70 (51.1)	
Medication usage, *n* (%)				
csDMARD	145 (80.6)	31 (72.1)	114 (83.2)	0.124
bDMARD	78 (43.3)	17 (39.5)	61 (44.5)	0.601
Prednisolone	33 (18.3)	7 (16.3)	26 (18.9)	0.823
NSAID daily	28 (15.6)	2 (4.7)	26 (19.0)	0.028
RA disease activity (Rapid 3)^j^, *n* (%)				
Near remission	59 (32.8)	21 (48.8)	38 (27.7)	0.003^e^
Low	39 (21.7)	11 (25.6)	28 (20.4)	
Moderate	71 (39.4)	9 (20.9)	62 (45.3)	
High	11 (6.1)	2 (4.7)	9 (6.6)	
Fatigue, mean (s.d.)^k^	4.98 (3.0)	4.14 (2.9)	5.25 (3.0)	0.031^c^

a Achieving 600 METs/week and completing two activities per week classified as muscle resistance activity.

b Chi-squared (exact), except where indicated.

c *t*-test.

d Home duties, student, caregiver, unemployed, other.

e Jockheere–Terpstra test for trend (exact).

f Reading difficulty or poor health literacy as indicated by the single item literacy screener.

g Four missing results for this variable: one missing from meeting guidelines and three missing from not meeting guidelines.

h England BR, Sayles H, Mikuls TR, Johnson DS, Michaud K. validation of the rheumatic disease comorbidity index. Arthritis Care Res 2015;67:865–72.

i Mann–Whitney U test.

j Hendrikx J, de Jonge MJ, Fransen J, Kievit W, van Riel PLCM. Systematic review of patient-reported outcome measures (PROMs) for assessing disease activity in rheumatoid arthritis. RMD Open 2016;2:e000202.

k Score range 0–10.

### Physical activity

**Figure 1.**
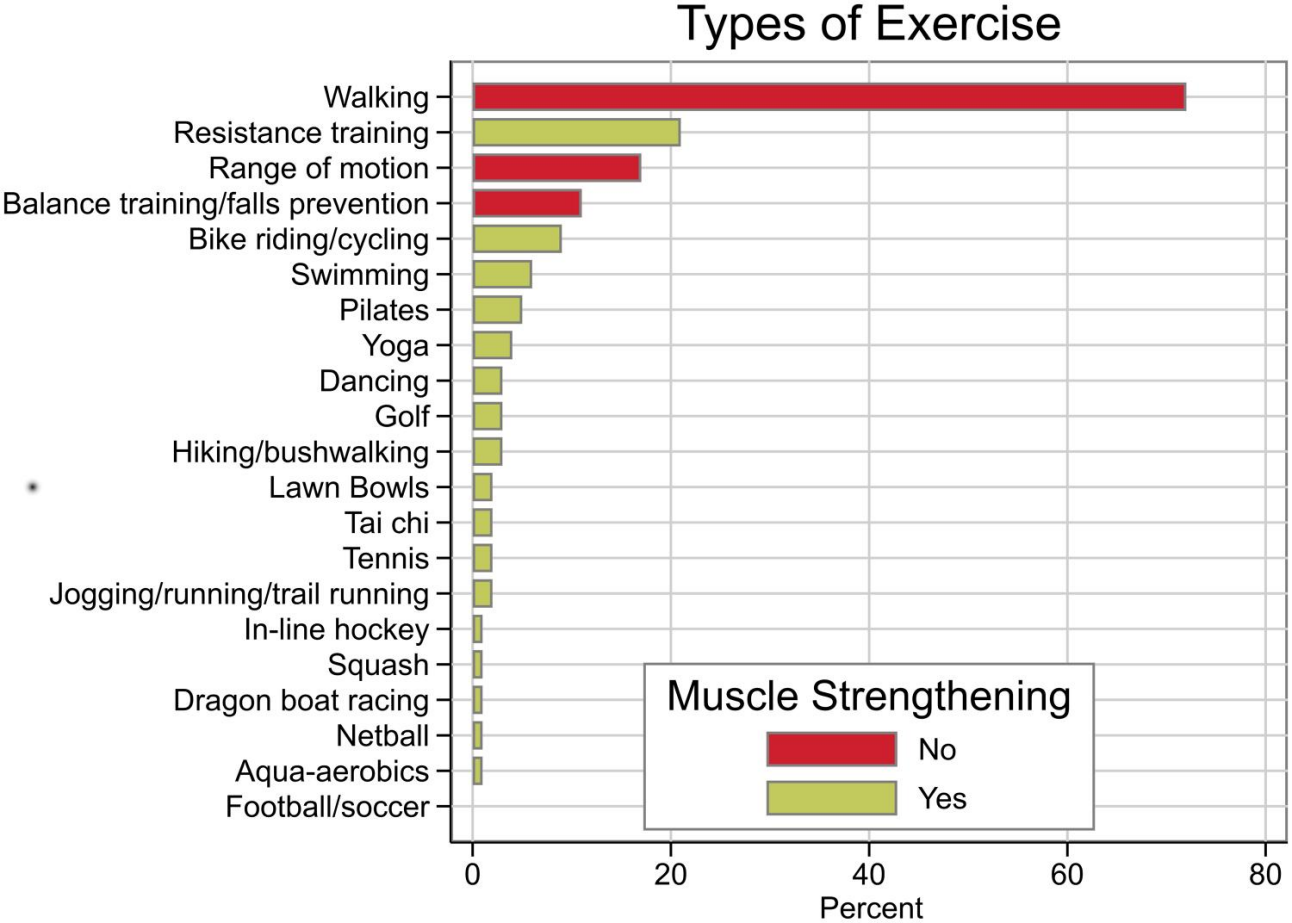
Self-reported physical activity

Walking was the preferred activity for most participants and, of the muscle strengthening activities, the most commonly reported were weight/bodyweight exercises, followed by cycling and swimming (Fig. 1).

Most participants (67%) expended >600 METs/week, but only 29% participated in muscle strengthening activities for two or more sessions per week for >10 min/session. About a quarter (24%) of participants met both criteria for the PA guidelines (‘meeting guidelines’ *vs* ‘not meeting guidelines’).

The differences in demographics and health status between those who did and did not meet the PA guidelines are presented in Table 1. Those who met guidelines were significantly more likely to have been recruited from a private *vs* public clinic and less likely to be overweight or obese, current smokers, use daily NSAIDs or have high disease activity, and reported less fatigue.

### Enablers and barriers to physical activity

**Figure 2.**
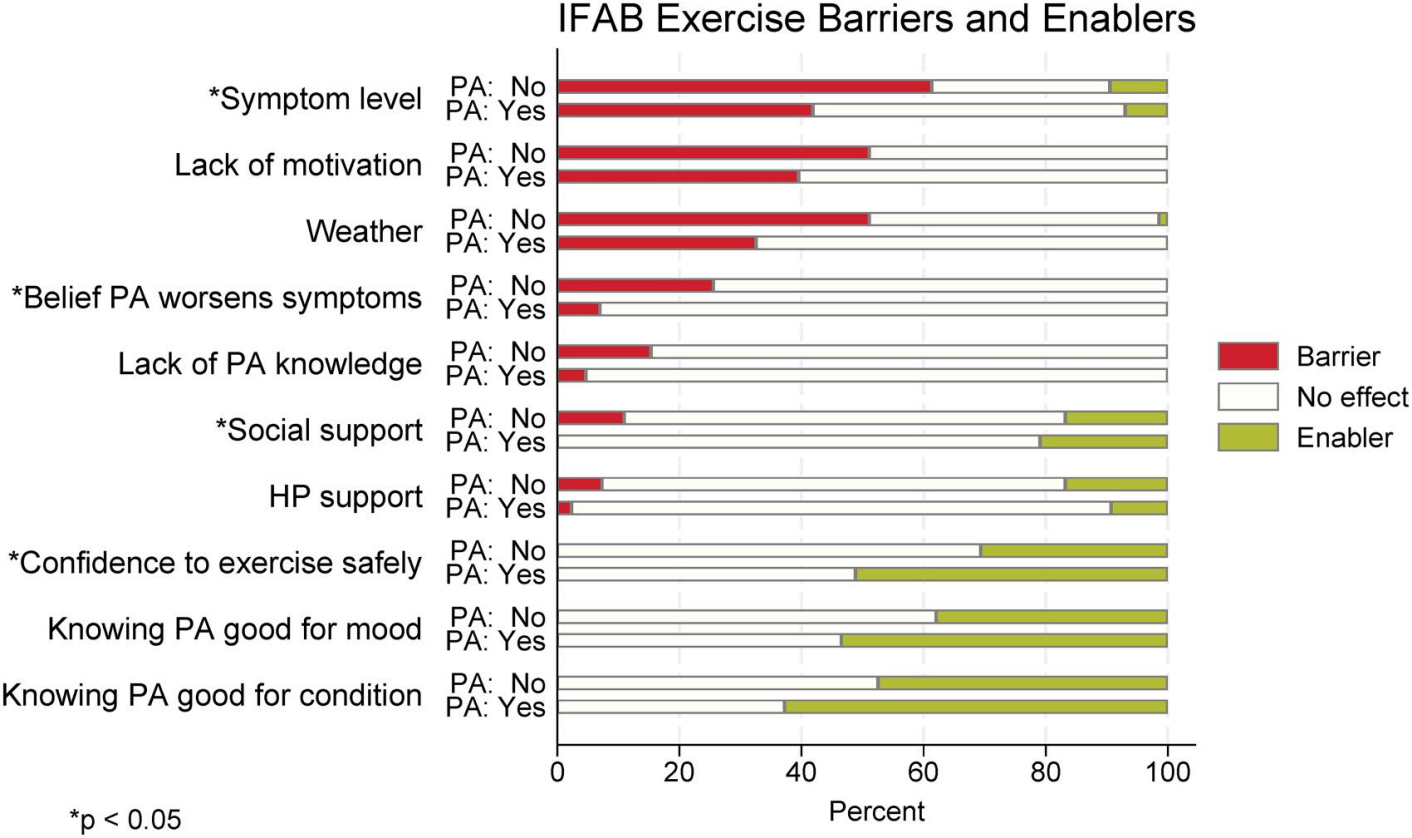
Proportion reporting barriers and enablers in the IFAB score. PA: physical activity; HP: health practitioner; PA: No: not meeting physical activity guidelines; PA: Yes: meeting physical activity guidelines

The most commonly reported barriers to PA were the presence of RA symptoms, lack of motivation and weather conditions (Fig. 2). The most commonly reported enablers included the knowledge that PA is good for RA, the knowledge that PA improves mood and feeling confident in knowing how to participate safely in PA.

A lower proportion of participants who met guidelines reported symptoms as a barrier compared with those who did not meet the guidelines. Those who met guidelines also were less likely to believe that PA worsened symptoms and had more confidence in their ability to exercise safely (Supplementary Table S2).

Overall, the total IFAB score was higher in those who met the PA guidelines compared with those who did not (Table 2). They also reported less impact from a lack of knowledge regarding what exercises to do and a greater impact from confidence in knowing how to exercise safely.

**Table 2:** Impact of barriers and enablers per the IFAB score.

Item (score range)	Score range	Whole group, mean (s.d.)	Meeting PA guidelines^a^, mean (s.d.) (*n* = 43)	Not meeting PA guidelines, mean (s.d.) (*n* = 137)	*P*-value^b^
Total IFAB (−70–70)^c^	−70–70	−1.86 (16.2)	6.62 (15.0)	−4.54 (15.7)	<0.001
Symptoms	−10–10	6.6 (2.5)	6.1 (2.2)	6.7 (2.5)	0.27
Weather	−10–10	5.6 (2.5)	5.1 (3.1)	5.7 (2.6)	0.56
Support from friends and family	−10–10	5.2 (2.65)	5 (2.9)	5.2 (2.6)	0.85
Support from healthcare	−10–10	6.1 (2.6)	7 (1.9)	5.9 (2.7)	0.31
A belief that PA will worsen symptoms	−10–0	−6.71 (2.6)	−5.7 (1.9)	−6.8 (2.6)	0.49
Lack of motivation	−10–0	−5.7 (2.8)	−5.8 (2.7)	−5.7 (2.8)	0.98
Lack of knowledge re what exercises to do	−10–0	−5.3 (2.7)	−4 (0)	−5.5 (2.8)	0.03
Knowledge that PA good for condition	0–10	6.1 (2.7)	6.5 (2.5)	5.9 (2.8)	0.31
Knowledge PA good for mood	0–10	5.8 (2.8)	6.7 (2.5)	5.4 (2.8)	0.05
Confidence knowing how to exercise safely	0–10	6.7 (2.7)	7.5 (1.8)	6.2 (2.9)	0.04

a Achieving 600 METs/week and completing two activities per week classified as muscle resistance activity.

b *t*-test.

c Negative scores reflect more barriers to physical activity and positive scores reflect fewer barriers and more enablers.

#### Health practitioner support

Of the 180 participants, 61.7% reported having discussed PA with their rheumatologist (Table 3). A statistically significantly higher proportion of those who met the PA guidelines reported discussing PA with their rheumatologist (66.7% *vs* 51.3%; *P* = 0.02) and finding the information from their healthcare team useful (65.1% *vs* 40.2%; *P* < 0.001).

**Table 3:** Healthcare support and physical activity beliefs.

Support/belief	Total (*N* = 180), *n* (%)	Meeting PA guidelines^a^ [*n* = 43 (24%)], *n* (%)	Not meeting PA guidelines [*n* = 137 (76%)], *n* (%)	*P*-value^b^
PA discussed with rheumatologist	111 (61.7)	33 (76.7)	78 (56.9)	0.02
Rheumatologist has ever recommended type or amount of PA	65 (58.6)	19 (57.6)	46 (59.0)	0.68
Enough PA information provided by rheumatologist	62 (55.9)	22 (66.7)	40 (51.3)	0.38
PA discussed with other health care team members	84 (46.7)	22 (51.2)	62 (45.3)	0.60
Enough information regarding PA provided by healthcare team	56 (67.5)	16 (76.2)	40 (64.5)	0.55
Information regarding PA provided by healthcare team has been useful	83 (46.1)	28 (65.1)	55 (40.2)	0.0004
Tampa Kinesiophobia Scale score^d^, mean (s.d.)	38.2 (7.8)	35.1 (8.1)	39.2 (7.5)	0.004^c^
Feels confident participating in PA	131 (72.8)	39 (90.7)	92 (67.2)	0.003
How important do you consider PA to your overall well-being? (0 = not important, 10 = very important)	8.45 (1.9)	9.2 (1.3)	8.2 (2.0)	0.001^c^
Believes the following PA recommendation is achievable:				
Adults should be active most days, preferably every day	139 (77.2)	33 (76.7)	106 (77.4)	0.9
Adults should do either 2.5-5 h of moderate-intensity PA or 1.25–2.5 h of vigorous-intensity PA or equivalent combination each week	101 (56.1)	33 (76.7)	68 (49.6)	0.004
Adults should include muscle strengthening activity at least 2 days each week	99 (55.0)	32 (74.4)	67 (48.9)	0.006
If >65 years of age, adults should do balance exercises 2–3 times a week	77/112 (42.8)	21 (48.9)	56 (40.9)	0.53

a Achieving 600 METs/week and completing two activities per week classified as muscle resistance activity.

b Chi-squared test (exact).

c *t*-test.

d Scores range from −17 to 68 (scores <37 are considered normal range and higher scores indicate increasing kinesiophobia).

The three most commonly reported sources of information regarding PA were physiotherapists (26.7%), general practitioners (24.4%) and online sources (24.4%). Participants reported that information should be received from their rheumatologist (47.5%), general practitioner (44.1%) and physiotherapist (37.9%) (Supplementary Table S3).

#### Beliefs regarding PA

Those who met PA guidelines reported a mean lower score on the Tampa Scale of Kinesiophobia compared with those who did not [35.1 (s.d. 8.1) *vs* 39.2 (s.d. 7.5); *P* = 0.004; Table 3]. Most participants (77.2%) agreed that it was achievable to be active on most days of the week. However, only 56.1% thought that it was possible to include the minimum recommended amount of moderate or vigorous intensity PA, and only 55% for strengthening activities (Table 3). Those who met the PA guidelines were more likely to believe it was possible to meet the recommendations for moderate to vigorous intensity (76.7% *vs* 49.6%; *P* = 0.004) and muscle strengthening (74.4% *vs* 48.9%; *P* = 0.006).

Overall, participants highly ranked the importance of exercise to their well-being, with a mean score of 8.45 (s.d. 1.9) on a scale from 0 (not important) to 10 (very important). Those who met PA guidelines ranked the importance of exercise higher than those who did not [9 (s.d. 1) *vs* 8 (s.d. 2); *P* = 0.001). Of the 180 participants, 72.8% reported feeling confident in participating in PA. However, most of these participants were in the group that met PA guidelines.

Most participants who did not meet the PA guidelines (*n* = 137) were either aware they were not meeting the recommendations or unsure (69%; Supplementary Table S4). Most of these participants indicated an intention to start exercising in the next 6 months, with 47% reporting an intention to increase their PA in the next month and 32% indicating an intention to increase their PA in the next 2–6 months.

#### Qualitative analysis

The following three themes were generated from the qualitative analysis: the relationship between PA and RA symptoms, the development of self-efficacy with ongoing PA and the hope for individualized and detailed advice. A description of key themes is included below (see Table 4 for quotes).

**Table 4:** Themes and subthemes generated from responses.

Themes	Subthemes	Examples of comments for each theme/subtheme	Demographics of commenter (age, gender, meeting/not meeting PA guidelines)
Symptom management	Exercise in the presence of symptoms requires modification of type, intensity and pacing	Too little (physical activity) is bad, but so is too much in my 55 years of experience living with it	74, female, no
		I am confident that where you have RA on your body makes a difference in what sort of exercise you undertake. I have RA in my ankles and I find swimming makes the ankles worse	56, female, no
		Understanding pace and peaks and troughs is critical	63, female, yes
		It does vary from day to day, and with age, so reasonable moderation and flexibility in expectations is necessary	63, male, no
		When you are unable to weight bear on hands due to inflammation in the wrists, push-ups, pull ups, weights and household tasks become impossible. Knee reconstruction surgery prevents squats/lunges so I struggle to find any muscle strengthening activity that I can do.	55, female, no
	Consistency is key	A little bit goes a long way. Consistency is key. Having daily movement is good. A couple of weeks ago I was able to do sprints on the rowing machine, but now as I’ve had a flare, I’ve had to talk myself out of giving up completely and sitting on the couch and doing nothing. Just because it doesn’t reach the guidelines, doesn’t mean it’s not worth doing. Even if it’s just a little walk down the street, it still helps	30, male, no
		I have been attending gym and doing resistant weight training, core exercises and stretching exercises for the last 20 years. I was diagnosed with RA approximately 35 years ago. I have found that exercising has made me stronger, improved my joints, and given me a better quality of life	77, female, yes
Self-efficacy with PA	Experience with exercise builds self-efficacy	I feel confident because I am comfortable to stop or modify an exercise depending on how I feel	50, female, no
	Prior bad experience can be a strong barrier	I hurt my back once and needed IV morphine and that has scared me from doing too much. I need to be able to look after my house and look after my garden. The acute pain after my back injury affected my independence, so that scared me. There’s no one to help me if anything goes wrong, so I’m very careful	66, female, no
	Gentle introduction more likely to be successful	It’s important for each individual to know their limitation and only do what they can do at the start of any exercise program. And to enjoy it most of all there is no point in over doing it and not enjoy it. No matter who you are or what medical problem you have you have to want to exercise to be of any benefit	62, female, no
Individualised and detailed advice	Concern regarding safety of advice in the setting of comorbidity	Would help to know I’m not putting myself at risk	58, female, yes
	Concerns regarding safety of exercise on joints	I would like more clarification on what kind/how much exercise I can do without damage to my joints	70, female, no
		I’m not sure if lifting and carrying things are good for our joints	58, female, no
	Further details wanted when provided exercise advice in a healthcare setting	I did ask a podiatrist once about exercises to strengthen ankles and he said there were but didn’t proceed to give me the exercises. I have had the condition for a long time and understand what I need to do to maintain things, and I know that regular exercise does keep the joints healthy	30, male, no
	Healthcare professionals to understand limitations in setting of flare/arthritis for clients to feel safe	Exercise is helpful if it can be done at a pace/style that allows me to adjust as I need. Sometimes it’s the pain afterwards that is a bigger problem than the pain during. I’m ok with adding a little pain at times in order to be able to do exercise/activities, but I do not want to be expected to put up with increased pain all the time when I can avoid excess pain by resting/taking things easy/slowly	50, female, no
		I think it is important that medical professionals do not make patients feel guilty (this has never been the case for me), as many people are just doing the best they can.	69, female, yes
	Supervised exercise needed by some but limited access	What is the best type of exercise for me personally? Note wanted a health care plan from my general practitioner but would have had to pay for a further appointment and I can’t afford it	66, female, no

### Symptom management

The first theme refers to a relationship with symptoms of inflammatory arthritis and physical activity. Attitudes varied between participants, with some suggesting that symptoms rendered physical activity impossible, while others viewed it as feasible with modification. Another pattern identified was that consistency with physical activity was associated with well-being.

### Self-efficacy with PA

This second theme refers to the development of self-efficacy in modifying activity. There were comments related to ‘knowing your limits’, as bad experiences were particularly detrimental to a person’s relationship with efficacy. A gentle introduction to physical activity was identified as a way people could become more confident in their own modifications.

### Individualised and detailed advice

This third theme highlighted concerns regarding the safety of exercise on the joints, and in the setting of comorbidities. While general physical activity advice may have been provided by healthcare professionals, more specific information about how to achieve that was lacking, leading to uncertainty. A few participants expressed that they would like to know the ‘best’ exercise for them. Perhaps indicating their understanding that working towards improving an element of physical fitness may not be as important as the ‘type’ of exercise itself. On the flipside, some highlighted concerns about accessibility/affordability after finding suitable options.

Overall, themes were related with a common underlying concern: ‘Is this safe for me?’. Participants who were able to modify intensity and activity type depending on symptoms expressed confidence in their ability to exercise safely.

## Discussion

In our study, 76% of people with RA were not meeting PA guidelines, and most did not believe it was possible to meet the guidelines in relation to the inclusion of muscle strengthening activity or more vigorous activity. The frequency and impact of barriers and enablers differed between those that did and did not meet the PA guidelines. Those who met PA guidelines had lower disease activity and were less likely to report RA symptoms as a barrier to PA. They felt more confident exercising safely, were more likely to have received useful advice from their healthcare team and reported less kinesiophobia. Safety concerns around physical activity were also highlighted in the qualitative analysis.

Only 24% of people with RA were meeting the national PA guidelines. While more than half were meeting the minimum number of minutes of PA recommended each week, most are not including muscle strengthening activities. In our study, 67% of participants expended >600 METs/week—comparable to previous studies by Demmelmaier *et al.* (69%) [16] and Manning *et al.* (61% >500 METs/week) [27]. Consistent with previous research, most participants in our study are meeting the recommended number of minutes of PA per week.

We found that 29% reported muscle strengthening activity, including 21% engaging in specific exercises (e.g. push-ups, free weights), a higher rate than in two previous RA studies. Demmelmaier *et al.* [16] reported 14% of participants reported strengthening activity twice a week, based on a modified question of the Exercise Stage Assessment Index. Iverson *et al.* [28] reported only 1% of their participants undertook weight training. However, our study was generous with the definition of strengthening activity, which may partly contribute to the differences in results. We included activities (other than weights) that have been shown to strengthen muscle and activities that could ‘potentially’ strengthen muscle if performed with sufficient intensity and frequency [29, 30]. While this method of measuring muscle strengthening activities has face validity, it was chosen as it allowed a comparison with the general Australian population. Using the same criteria, 53.7% (95% CI 52.8, 54.6) of Australians ages 18–64 years reported muscle strengthening activity (2017–2018 Australian Institute of Health and Welfare analysis of AusPlay data) [25]. This is higher than for the RA participants in this study. Differences may be partially attributed to older age of RA participants or differences in data collection (AusPlay used phone interviews, while our study used self-administered questionnaires). Given the minority of people with RA engaged in muscle strengthening activities, and the role muscle strengthening plays in bone health, which is a common comorbidity in RA, this should be a focus for health promotion. Walking on flat ground is not considered a muscle strengthening activity; however, walking was the most popular choice of PA in our study, consistent with Iverson *et al.* [28], highlighting a potential area for modification.

A limitation of our study is the potential overestimation of PA participation due to self-selection bias from our recruitment strategy, which may have attracted more active individuals, and the social desirability bias inherent in self-reported measures like the IPAQ. There is only fair to moderate agreement between the IPAQ-SF and accelerometry [22]. The use of objective measures would reduce these biases [31]. Another limitation of our study was recruitment from largely metropolitan areas in South Australia, limiting generalisability to rural and remote areas.

The most commonly reported barrier to PA was symptoms of RA. Notably, those who met the PA guidelines were less likely to have high disease activity and were less likely to consider symptoms a barrier. This finding was supported by our qualitative analysis of the open-ended questions in our questionnaire, where the relationship between physical activity and RA symptoms were identified as a key theme. This is in line with a recent qualitative meta-synthesis that found RA acted as a persistent catalyst, either as a barrier or enabler of PA [32]. Previous studies have also shown lower PA uptake in those with higher disease activity [14, 33]. However, Toyoshimo *et al.* [34] did not find an association once corrected for fatigue. Given the cross-sectional nature of our study, we are unable to comment on the direction of the temporal relationship between PA and disease activity. Comments made by participants regarding flares of symptoms related to higher levels of PA highlight this as an area for further research.

Despite the majority of people not meeting the PA guidelines, our participants highly rated the importance of PA. Knowledge of the benefits of PA for mood and the confidence to exercise safely were reported more commonly and with greater impact in the group that met PA guidelines. This group was also more likely to have discussed PA with a rheumatologist. However, our finding that most people did not believe it was possible to meet the guidelines regarding muscle strengthening activity is novel, and significant in highlighting a potential area for education and change in practice.

Specific and practical advice regarding how muscle strengthening activity can be gradually added into everyday or recreational activity is a potential area for focus, as is how we can help people with RA participate in PA safely and with confidence. Advice from rheumatology clinicians—particularly when personalised and addressing safety and disease-specific concerns—has been shown to improve physical activity participation in people with RA [16, 35, 36].

The need for more detailed physical activity advice from health practitioners was identified as a key theme in our thematic analysis. This is in-line with previous qualitative research in this area. Thomsen *et al.* [37] found that the topic of PA was addressed sporadically by health professionals in clinics, and mostly at the request of the individual patient, and that the quality of the advice when received was varied. Larkin *et al.* [38] also highlighted practitioner uncertainty and variation in recommendations, particularly in the setting of a flare, and recommended further work in this area to help guide clinicians.

These findings highlight that most people with RA do not think it is possible to engage in regular muscle strengthening activities or exercise of moderate to vigorous intensity. Disease activity was associated with lower PA uptake. While tight control of RA is paramount for the rheumatologist, there is also a role here potentially for a wider public health approach and on an individual-level for engaging allied health support to help provide education regarding the various ways strengthening activity and higher intensities may be introduced safely into regular programs. Swärdh *et al.* [32] explore the range of roles a physical therapist plays in the promotion of PA in rheumatic diseases, from education to developing self-efficacy to allow participants autonomy outside the healthcare system. Further work is needed to explore how PA in RA is addressed by different healthcare providers in the Australian context and how we provide services over a range of healthcare disciplines.

A key strength of this study is its comprehensive evaluation of both aerobic and muscle strengthening physical activity. The definitions used were aligned with Australian national guidelines. The study also explored modifiable factors that influence PA participation, such as confidence, perceptions of safety and professional advice. This provides useful insights to help design interventions to support PA in people with RA.

## Supplementary Material

rkaf146_Supplementary_Data

## Data Availability

The data underlying this article cannot be shared publicly due to participant privacy. The data will be shared on reasonable request to the corresponding author.
