# Supplementary material for: Understanding physical activity participation in people living with rheumatoid arthritis
Source: Rheumatol Adv Pract. 2025 Dec 23;10(1):rkaf146. doi: 10.1093/rap/rkaf146 (PMC12798537; doi:10.1093/rap/rkaf146)
Supplement: rkaf146_Supplementary_Data [file rkaf146_supplementary_data.docx]

**Supplementary material S1: Questionnaire**

1. **Age**: _________

1. **Gender:**

☐ Female

☐ Male

☐ Non-binary

1. **Address***: __________________________________

**All information will be de-identified and coded; however, if you would prefer not to use your full address, you can list your postcode instead.*

1. **Work Status:**

☐ Full-time employment

☐ Part-time employment

☐ Student

☐ Retired

☐ Home duties

☐ Carer

☐ Unemployed

☐ Other

1. **What is your highest level of education?**

☐ Didn't finish high school

☐ Finished high school

☐ Trade/Apprenticeship

☐ Certificate/Diploma

☐ Bachelor degree or higher

1. **What is the main language you speak at home?**

☐ English

☐ Italian

☐ Mandarin

☐ Greek

☐ Vietnamese

☐ Cantonese

☐ Punjabi

☐ Arabic

☐ Hindi

☐ German

☐ Polish

☐ Spanish

☐ other

☐ If "other" language, please specify __________________________________

1. **How often do you need to have someone help you when you read instructions, pamphlets, or other written material from your doctor or pharmacy?**

☐ Never

☐ Rarely

☐ Sometimes

☐ Often

☐ Always

1. **What is your approximate height?**  _________cm **OR** _________ feet/inches

**9.What is your approximate weight?** _________ kg

1. **Are you a current smoker?**

☐ Yes

☐ No, never

☐ No, ex-smoker

1. **As far as you know, do you have, or have you had, any of the health conditions that are listed below?**

Please tick all that apply.

| **Condition** | **Now** |  | **In the past** |
| --- | --- | --- | --- |
| High blood pressure | ☐ |  | ☐ |
| Heart attack | ☐ |  | ☐ |
| Other heart conditions | ☐ |  | ☐ |
| Stroke | ☐ |  | ☐ |
| Depression | ☐ |  | ☐ |
| Mental illness | ☐ |  | ☐ |
| Diabetes | ☐ |  | ☐ |
| Cancer | ☐ |  | ☐ |
| Alcohol or drug problem | ☐ |  | ☐ |
| Kidney problem | ☐ |  | ☐ |
| Lung problem | ☐ |  | ☐ |
| Asthma | ☐ |  | ☐ |
| Cataract | ☐ |  | ☐ |
| Severe allergies | ☐ |  | ☐ |
| Liver problem | ☐ |  | ☐ |
| Gall bladder problem | ☐ |  | ☐ |
| Ulcer | ☐ |  | ☐ |
| Other stomach problems | ☐ |  | ☐ |
| Neurological problems (like seizures, Parkinson’s disease, multiple sclerosis etc) | ☐ |  | ☐ |
| Fractures of the spine/hip or leg | ☐ |  | ☐ |
| Thyroid or endocrine disorder | ☐ |  | ☐ |
| Problems with prostate (men) or uterus, ovaries, etc (women) | ☐ |  | ☐ |
| None of the above |  | ☐ |  |

1. **What year (approximately) were you diagnosed with rheumatoid arthritis*?***

*_________*

1. **Please tick which of the following medication(s) you are currently on:**

☐ Methotrexate *(Methoblastin/Trexject)*

☐ Leflunomide *(Arava, Arabloc)*

☐ Sulfasalazine *(Pyralin EN, Salazopyrin EN)*

☐ Hydroxychloroquine *(Plaquenil)*

☐ Abatacept *(Orencia)*

☐ Adalimumab *(Humira, Amgevita, Hadlima, Hyrimoz, Idacio, Abrilada, Hulio)*

☐ Baricitinib *(Olimiant)*

☐ Certolizumab *(Cimzia)*

☐ Etanercept *(Enbrel, Brenzys, Erelzi, Etera, Rymti)*

☐ Golimumab *(Simponi)*

☐ Infliximab *(Remicade, Inflectra, Renflexis)*

☐ Rituximab *(MabThera, Riximyo, Rixonfya, Rixvyda, Truxima, Ritemvia, Rituzena, Tuxella, Ruxience)*

☐ Tocilizumab *(Actemra)*

☐ Upadacitinib *(Rinvoq)*

☐ None of the above

1. **Do you take non-steroidal anti-inflammatory medication (NSAIDs)?**

☐ No

☐ Yes - Daily

☐ Yes – intermittently

1. **Regarding prednisolone, which best describes your situation:**

☐ Have never used

☐ Currently taking

☐ Not currently taking, but used within the last 6 months

☐ Not currently taking, last used more than 6 months ago

1. **Regarding function and pain:**

Please answer the following questions which are meant to assess how active your arthritis is at this time.

Over the last week I was able to…

|  | Without any  difficulty | With some  difficulty | With much  difficulty | Unable to do |
| --- | --- | --- | --- | --- |
| Dress myself including shoelaces and buttons. | ☐ | ☐ | ☐ | ☐ |
| Get in and out of bed. | ☐ | ☐ | ☐ | ☐ |
| Lift a full cup to my mouth. | ☐ | ☐ | ☐ | ☐ |
| Walk outdoors on flat ground. | ☐ | ☐ | ☐ | ☐ |
| Wash and dry my entire body. | ☐ | ☐ | ☐ | ☐ |
| Bend down to pick up clothing off of the floor. | ☐ | ☐ | ☐ | ☐ |
| Turn regular faucets on and off. | ☐ | ☐ | ☐ | ☐ |
| Get in and out of the car, bus, train, or airplane. | ☐ | ☐ | ☐ | ☐ |
| Walk two miles, or three kilometers if I wish. | ☐ | ☐ | ☐ | ☐ |
| Participate in recreational sports and activities if I wish. | ☐ | ☐ | ☐ | ☐ |
| Get a good night’s sleep | ☐ | ☐ | ☐ | ☐ |
| Deal with feelings of anxiety or feeling nervous | ☐ | ☐ | ☐ | ☐ |
| Deal with feelings of depression or feeling blue | ☐ | ☐ | ☐ | ☐ |

How much pain did you have because of your condition over the last week?

Please indicate how severe your pain has been.

| No pain | 0 | 1 | 2 | 3 | 4 | 5 | 6 | 7 | 8 | 9 | 10 | Pain as bad as it could be |
| --- | --- | --- | --- | --- | --- | --- | --- | --- | --- | --- | --- | --- |
|  | ☐ | ☐ | ☐ | ☐ | ☐ | ☐ | ☐ | ☐ | ☐ | ☐ | ☐ |  |

Considering all the ways in which illness and health conditions may affect you at this time, please indicate how you are doing (0 is very well, 10 is very poorly).

| Very well | 0 | 1 | 2 | 3 | 4 | 5 | 6 | 7 | 8 | 9 | 10 | Very poorly |
| --- | --- | --- | --- | --- | --- | --- | --- | --- | --- | --- | --- | --- |
|  | ☐ | ☐ | ☐ | ☐ | ☐ | ☐ | ☐ | ☐ | ☐ | ☐ | ☐ |  |

1. **How much of a problem has fatigue or tiredness been for you over the past week?**

| No problem | 0 | 1 | 2 | 3 | 4 | 5 | 6 | 7 | 8 | 9 | 10 | Major problem |
| --- | --- | --- | --- | --- | --- | --- | --- | --- | --- | --- | --- | --- |
|  | ☐ | ☐ | ☐ | ☐ | ☐ | ☐ | ☐ | ☐ | ☐ | ☐ | ☐ |  |

1. **Do you think you do enough physical activity?**

☐ Yes → If Yes, continue to question 19.

☐ No → If No, continue to question 20.

☐ Unsure → If unsure, continue to question 20

1. **Have you been taking part in regular physical activity for more than 6 months?** ☐Yes

☐No

1. **Do you intend to start exercising regularly?**

☐No

☐Yes, in the next month

☐Yes, probably not in the next month, but in the next 6 months

☐ N/A I already exercise regularly

1. **International Physical Activity Questionnaire**

We are interested in finding out about the kinds of physical activities that people do as part of their everyday lives. The questions will ask you about the time you spent being physically active in the **last 7 days**. Please answer each question even if you do not consider yourself to be an active person. Please think about the activities you do at work, as part of your house and yard work, to get from place to place, and in your spare time for recreation, exercise or sport.

Think about all the **vigorous** activities that you did in the **last 7 days**. **Vigorous** physical activities refer to activities that take hard physical effort and make you breathe much harder than normal. Think *only* about those physical activities that you did for at least 10 minutes at a time.

1. During the **last 7 days**, on how many days did you do **vigorous** physical activities like heavy lifting, digging, aerobics, or fast bicycling?

_____ **days per week**

No vigorous physical activities  ***Skip to question (3)***

1. How much time did you usually spend doing **vigorous** physical activities on one of those days?

_____ **hours per day**

_____ **minutes per day**

Don’t know/Not sure

Think about all the **moderate** activities that you did in the **last 7 days**. **Moderate** activities refer to activities that take moderate physical effort and make you breathe somewhat harder than normal. Think only about those physical activities that you did for at least 10 minutes at a time.

1. During the **last 7 days**, on how many days did you do **moderate** physical activities like carrying light loads, bicycling at a regular pace, or doubles tennis? Do not include walking.

_____ **days per week**

No moderate physical activities  ***Skip to question (5)***

1. How much time did you usually spend doing **moderate** physical activities on one of those days?

_____ **hours per day**

_____ **minutes per day**

Don’t know/Not sure

Think about the time you spent **walking** in the **last 7 days**. This includes at work and at home, walking to travel from place to place, and any other walking that you have done solely for recreation, sport, exercise, or leisure.

1. During the **last 7 days**, on how many days did you **walk** for at least 10 minutes at a time?

_____ **days per week**

No walking ***Skip to question 7***

1. How much time did you usually spend **walking** on one of those days?

_____ **hours per day**

_____ **minutes per day**

Don’t know/Not sure

1. **Type of physical activity for recreation**

The next questions ask about the type of physical activity you do for recreation, leisure or fitness. It does not include activity for work or transport or housework.

If you do not do any physical activity for recreation, leisure or fitness, you may skip this section.

**Please indicate if you have taken part in the below exercises in the last week or if not in the last week, in the last month.**

| **Activities** | **Yes** | **Number of times per week** | **If less regularly than weekly, number of times per month** | **Approximate time spent on an average session (minutes)** |
| --- | --- | --- | --- | --- |
| Walking | ☐ |  |  |  |
| Hiking/bushwalking | ☐ |  |  |  |
| Jogging/running/trail running | ☐ |  |  |  |
| Swimming | ☐ |  |  |  |
| Bike riding or cycling | ☐ |  |  |  |
| Yoga | ☐ |  |  |  |
| Pilates | ☐ |  |  |  |
| Muscle strengthening exercises Examples include :  Body weight exercises like push ups/pull ups  Exercises with weights – free weights  and machine weights | ☐ |  |  |  |
| Tennis | ☐ |  |  |  |
| Football/soccer | ☐ |  |  |  |
| Golf | ☐ |  |  |  |
| Balance training/falls prevention exercises | ☐ |  |  |  |
| Stretching exercises | ☐ |  |  |  |
| Thai chi | ☐ |  |  |  |
| Dancing | ☐ |  |  |  |

**If you take part in a sport or exercise regularly that is not listed above, you may write in in the columns below.**

| **Activities** | **Number of times per week** | **If less regularly than weekly, number of times per month** | **Approximate time spent on an average session**  **(minutes)** |
| --- | --- | --- | --- |
|  |  |  |  |
|  |  |  |  |
|  |  |  |  |

1. **Barriers and Facilitators**

Please take some time to think about all the physical activity you did in the previous month: walking, jogging, gardening, other kind of sport…

Now, think about all the things that have encouraged you, and all the things that prevented you form doing physical activity in the previous month.

This questionnaire aims to collect all the things that have encouraged you or prevented you from doing physical activity in the previous month.

**Please indicate for each item if it has rather encouraged you, prevented you, or had no impact on your physical activity in the previous month (only one answer). If needed, rate the importance.**

- 1. **Level of symptoms (pain, fatigue, lack of mobility)**

 prevented me from doing physical activity in the previous month

 encouraged to do physical activity in the previous month

 had no impact on my physical activity in the previous month

| If this item impacted your involvement in physical activity, what was the impact? |
| --- |
|  |
| **Had no impact on my Had a maximal impact on**  **0 1 2 3 4 5 6 7 8 9 10 physical activity my physical activity** |

- 1. **Weather conditions**

 prevented me from doing physical activity in the previous month

 encouraged to do physical activity in the previous month

 had no impact on my physical activity in the previous month

| If this item impacted your involvement in physical activity, what was the impact? |
| --- |
|  |
| **Had no impact on my Had a maximal impact on**  **0 1 2 3 4 5 6 7 8 9 10 physical activity my physical activity** |

- 1. **Presence or absence of support from others (friends, family)**

 prevented me from doing physical activity in the previous month

 encouraged to do physical activity in the previous month

 had no impact on my physical activity in the previous month

| If this item impacted your involvement in physical activity, what was the impact?  **Had no impact on my Had a maximal impact on**  **0 1 2 3 4 5 6 7 8 9 10 physical activity my physical activity** |
| --- |

- 1. **Presence or absence of support and/or advice from healthcare professionals**

 prevented me from doing physical activity in the previous month

 encouraged to do physical activity in the previous month

 had no impact on my physical activity in the previous month

| If this item impacted your involvement in physical activity, what was the impact? |
| --- |
|  |
| **Had no impact on my Had a maximal impact on**  **0 1 2 3 4 5 6 7 8 9 10 physical activity my physical activity** |

- 1. **A belief that physical activity will make my symptoms worse**

 prevented me from doing physical activity in the previous month

 had no impact on my physical activity in the previous month

| If this item impacted your involvement in physical activity, what was the impact? |
| --- |
|  |
| **Had no impact on my Had a maximal negative**  **physical activity 0 1 2 3 4 5 6 7 8 9 10 impact on my physical**  **activity** |

- 1. **Lack of motivation**

 prevented me from doing physical activity in the previous month

 had no impact on my physical activity in the previous month

| If this item impacted your involvement in physical activity, what was the impact? |
| --- |
|  |
| **Had no impact on my Had a maximal negative**  **physical activity 0 1 2 3 4 5 6 7 8 9 10 impact on my physical**  **activity** |

- 1. **Lack of knowledge on which exercises to do and how much**

 prevented me from doing physical activity in the previous month

 had no impact on my physical activity in the previous month

| If this item impacted your involvement in physical activity, what was the impact? |
| --- |
|  |
| **Had no impact on my Had a maximal negative**  **physical activity 0 1 2 3 4 5 6 7 8 9 10 impact on my physical**  **activity** |

- 1. **Knowledge that physical activity is good for my condition**  encouraged to do physical activity in the previous month

 had no impact on my physical activity in the previous month

| If this item impacted your involvement in physical activity, what was the impact? |
| --- |
|  |
| **Had no impact on my Had a maximal positive**  **physical activity 0 1 2 3 4 5 6 7 8 9 10 impact on my physical**  **activity** |

**11. Knowledge of benefits of physical activity for mood**

 encouraged to do physical activity in the previous month

 had no impact on my physical activity in the previous month

| If this item impacted your involvement in physical activity, what was the impact? |
| --- |
|  |
| **Had no impact on my Had a maximal positive**  **physical activity 0 1 2 3 4 5 6 7 8 9 10 impact on my physical**  **activity** |

**10. Confidence that I know how to exercise safely**

 encouraged to do physical activity in the previous month

 had no impact on my physical activity in the previous month

| If this item impacted your involvement in physical activity, what was the impact? |
| --- |
|  |
| **Had no impact on my Had a maximal positive**  **physical activity 0 1 2 3 4 5 6 7 8 9 10 impact on my physical**  **activity** |

1. **Where do you get your information regarding physical activity? Tick all that apply.**

☐ No information

☐ Exercise physiologist

☐ GP

☐ Physiotherapist

☐ Rheumatologist

☐ Online

☐ Friends/family

☐ Other fitness professionals (eg personal trainer, gym instructor)

☐ Other __________________________________

1. **Where do you think you should be getting your information regarding physical activity from? Tick all that apply.**

☐ No preference

☐ Exercise physiologist

☐ GP

☐ Physiotherapist

☐ Rheumatologist

☐ Online

☐ Friends/family

☐ Other fitness professionals (eg personal trainer, gym instructor)

☐ Other __________________________________

1. **Have you found the information provided by your health care team regarding exercise useful?**

☐ Yes

☐ No

☐ Not sure

☐ No information given by health care team

1. **Have you ever discussed physical activity with your rheumatologist?**

☐ Yes

☐ No

(If No - continue to question 32)

1. **Has your rheumatologist ever recommended the type or amount of physical activity you should be doing?**

☐ Yes

☐ No

☐ Not sure

1. **Do you feel you have been given enough information regarding physical activity in rheumatoid arthritis by your rheumatologist?**

☐ Yes

☐ No

☐ Not sure

1. **Have you discussed physical activity with other members of your health care team?**

☐ Yes

☐ No (If no, continue to question 35.)

1. **If yes, can you list which health professionals have given you information**

|  |
| --- |

1. **Do you feel like you have been given enough information about physical activity by your health care team?**

☐ Yes

☐ No

☐ Not sure

1. **If you have not discussed PA with your rheumatologist or health care team, is there anything you would you like to find out?**

|  |
| --- |

1. **Do you feel confident participating in exercise?**

☐ Yes

☐ No

1. **Would anything help you feel more confident to participate in exercise?**

|  |
| --- |

1. **How important do you consider physical activity to your overall wellbeing?**

| Not important | 0 | 1 | 2 | 3 | 4 | 5 | 6 | 7 | 8 | 9 | 10 | Very important |
| --- | --- | --- | --- | --- | --- | --- | --- | --- | --- | --- | --- | --- |
|  | ☐ | ☐ | ☐ | ☐ | ☐ | ☐ | ☐ | ☐ | ☐ | ☐ | ☐ |  |

1. **Please answer the following questions according to your true feelings, not according to what others think you should believe. Score each statement from strongly disagree to strongly agree by tapping/circling the appropriate box**

|  | **Strongly disagree** | **Disagree** | **Agree** | **Strongly Agree** |
| --- | --- | --- | --- | --- |
| 1. I’m afraid that I might injury myself if I exercise | 1 | 2 | 3 | 4 |
| 2. If I were to try to overcome it, my pain would increase | 1 | 2 | 3 | 4 |
| 3. My body is telling me I have something dangerously wrong | 1 | 2 | 3 | 4 |
| 4. My pain would probably be relieved if I were to exercise | 1 | 2 | 3 | 4 |
| 5. People aren’t taking my medical condition seriously enough | 1 | 2 | 3 | 4 |
| 6. My accident *(or arthritis)* has put my body at risk for the rest of my life | 1 | 2 | 3 | 4 |
| 7. Pain always means I have injured my body | 1 | 2 | 3 | 4 |
| 8. Just because something aggravates my pain does not mean it is dangerous | 1 | 2 | 3 | 4 |
| 9. I am afraid that I might injure myself accidentally | 1 | 2 | 3 | 4 |
| 10. Simply being careful that I do not make any unnecessary movements is the safest thing I can do to prevent my pain from worsening | 1 | 2 | 3 | 4 |
| 11. I wouldn’t have this much pain if there weren’t something potentially dangerous going on in my body | 1 | 2 | 3 | 4 |
| 12. Although my condition is painful, I would be better off if I were physically active | 1 | 2 | 3 | 4 |
| 13. Pain lets me know when to stop exercising so that I don’t injure myself | 1 | 2 | 3 | 4 |
| 14. It’s really not safe for a person with a condition like mine to be physically active | 1 | 2 | 3 | 4 |
| 15. I can’t do all the things normal people do because it’s too easy for me to get injured | 1 | 2 | 3 | 4 |
| 16. Even though something is causing me a lot of pain, I  don’t think it’s actually  dangerous | 1 | 2 | 3 | 4 |
| 17. No one should have to exercise when he/she is in pain | 1 | 2 | 3 | 4 |

1. **The following statements relate to some recommendations about exercise in rheumatoid arthritis. In the tick boxes next to each statement, please indicate whether you think this is achievable in your day-to-day life?**

| **Recommendation** | **I think this is achievable for me :** | |  |
| --- | --- | --- | --- |
|  | **Yes** | **No** | **Unsure** |
| Adults should be active most days ; preferably every day | ☐ | ☐ | ☐ |
| Each week ; adults should do either :   - 2.5-5 hours **moderate** intensity physical activity (such as a brisk walk, golf, mowing the lawn or swimming) - 1.25-2.5 hours **vigorous** intensity physical activity (such as jogging, aerobics, fast cycling, soccer or netball) - An equivilant combination of moderate and vigorous activities | ☐ | ☐ | ☐ |
| Include muscle strengthening activities on at least 2 days each week. This can be :   - push-ups - pull-ups - squats or lunges - lifting weights - household tasks that involve lifting, carrying or digging | ☐ | ☐ | ☐ |
| If you are <65 years old ; please go to **Q40.**  If you are 65 years of age or older ; balance exercises 2-3 times per week (for example tai chi and yoga) can help reduce falls | ☐ | ☐ | ☐ |

1. **Do you have any comments about these recommendations?**

|  |
| --- |

1. **Is there anything else that you would like to tell us about exercise and rheumatoid arthritis?**

|  |
| --- |

1. **How were you recruited for this survey?**

☐ Public hospital clinic

☐ Private rheumatology clinic

☐ Online

☐ Other

**Thank you for participating in this survey.**

**Supplementary Table S1: Factors in analysis**

| **Covariate** | **Description** | **Comments/references** |
| --- | --- | --- |
| **Demographics** |  |  |
| *Age (years)* | Continuous |  |
| *Gender* | Female, Male, Non-binary |  |
| *Socioeconomic status (SES)* | Quintiles ranging from 1 (highest SES) - 5 (lowest SES)  Address/postcode information used in Australian census interactive map (1) to determinate statistical area 1 (SA1) code.  SA1 classified into corresponding index of relative socioeconomic advantage or disadvantage (IRSAD).  IRSAD index (2) uses census variables related to SES status to classify Australian population into quintiles. | <https://www.abs.gov.au/ausstats/>  [abs@.nsf/Lookup/by%20Subject/](mailto:abs@.nsf/Lookup/by%20Subject/)  2033.0.55.001~2016~  Main%20Features~IRSAD%20  Interactive%20Map~16 |
| *Occupational status* | Full-time retirement, part-time retirement, student, retired, home-duties, carer, unemployed, other |  |
| *Highest level of education* | Didn’t finish high-school, finished high-school, trade/apprenticeship, certificate/diploma, bachelor degree or higher |  |
| *Language spoken at home* | English, Italian, Mandarin, Greek, Vietnamese, Cantonese, Punjabi, Arabic, Hindi, German, Polish, Spanish, other |  |
| *Health literacy* | Single Item Literacy Screener (SILS) | Performs well at identifying those with limited reading ability (3) |
| *Recruitment* | Public hospital clinic, private rheumatology clinic, online, other |  |
| **Health Status** |  |  |
| *Body Mass Index (BMI)* | Self-reported height and weight used to calculate BMI:  Underweight (<18.5), normal range (18.5-24.9), pre-obesity (25.0-29.9), obese (30+) | Calculation and classification as per World Health Organisation (4) |
| *Smoking status* | Yes, no- never, no-ex-smoker |  |
| *Co-morbidities* | Rheumatic Disease Comorbidity Index (RDCI) (5) calculated as per England et al **(6)**  Score (0-9)  Higher score indicating higher comorbidity burden | Validated in RA (6) |
| *RA disease duration* | Calculated from approximate year diagnosed, and classified as: 0-5 years, 6-10 years, >11 years |  |
| *Current medication use:* | List of conventional and biologic disease modifying agent shown vs none of the above  No NSAID use versus daily or intermittent use  Never used prednisolone versus currently taking, or not currently taking but last used either more or less than 6 months ago |  |
| *RA disease activity* | Rapid 3 assessment  Cumulative score (1-30) classified as: Near remission 1-3, Low severity (4-6), Moderate severity (7-12), High severity (13-30) | Validated in RA (7) |
| *Fatigue* | How much of a problem has fatigue or tiredness been for you over the past week?  0-10 scale (0=no problem, 10 = major problem) |  |
| **PA Enablers and Barriers** |  |  |
| *Enablers and barriers to PA* | - Inflammatory arthritis Facilitators and Barriers (IFAB) questionnaire includes ten items:   - Four items considered barriers or enablers (scored -10-10) including symptoms, weather, social support, and health professional support - Three items considered barriers (scored -10-0) including a belief that PA will make symptoms worse, lack of motivation, and lack of knowledge re: exercise - Three items considered enablers (scored 0-10) including the knowledge that PA is good for condition, mood and confidence to exercise safely   The following variables reported in our study:  - Frequency of barriers/enablers reported  - mean impact score of each item  -Total IFAB score reported (-70-70) with lower scores indicating more barriers, higher scores indicating more enablers | - Score developed based on commonly reported barriers and enablers (8) - Total IFAB score validated in RA (9) |
| *Kinesiophobia* | - Tampa scale of kinesiophobia-17 - Scores range from -17-68 (Scores below 37 are considered normal range, and higher score indicate increasing kinesiophobia (10) | Validated in musculoskeletal pain. (10) |
| *Where do you get your information regarding PA?* | - no information, exercise physiologist, general practitioner, rheumatologist, physiotherapist, online, friends/ family, other fitness professionals (personal trainer or gym instructor) or other |  |
| *Where do you think you should be getting your information regarding PA?* | - no information, exercise physiologist, general practitioner, rheumatologist, physiotherapist, online, friends/ family, other fitness professionals (personal trainer or gym instructor) or other |  |
| *Health professional support* | - Yes, No, Not sure, No information - Have you found the information provided by your health care team regarding exercise useful? - Have you ever discussed PA with your rheumatologist? - Have you ever discussed PA with other members of your health care team |  |
| **PA perceptions** |  |  |
| *Importance of PA to well-being* | Scale 0-10 (0=not important, 10 = very important) |  |
| *Confidence participating in PA* | Yes, no |  |
| *Achievability of PA guidelines* | Guidelines listed and following options provided: I think this is achievable /I do not think is achievable / unsure | Guidelines (11) |
| *Stage of behavioural change* | Maintenance, active, preparatory, contemplative and pre-contemplative stages | Based on the trans-theoretical model for behavioural change and stages of exercise scale (12, 13) |

1. IRSAD interactive map - Socio-Economic Indexes for Areas (SEIFA), Australia: Australian Bureau of Statistics; 2016 [Available from: <https://www.abs.gov.au/ausstats/abs@.nsf/Lookup/by%20Subject/2033.0.55.001~2016~Main%20Features~IRSAD%20Interactive%20Map~16>.

2. Technical Paper - Socio-economic indexes for areas (SEIFA) Canberra Australian Bureau of Statistics 2016 [Available from: <https://www.ausstats.abs.gov.au/ausstats/subscriber.nsf/0/756EE3DBEFA869EFCA258259000BA746/$File/SEIFA%202016%20Technical%20Paper.pdf>.

3. Morris NS, MacLean CD, Chew LD, Littenberg B. The Single Item Literacy Screener: Evaluation of a brief instrument to identify limited reading ability. BMC Fam Pract. 2006;7(1):21-.

4. Organisation. WWH. Obesity: preventing and managing the global epidemic: report of a WHO consultation. Geneva, Switzerland 2000.

5. Michaud K, Wolfe F. Comorbidities in rheumatoid arthritis. Best Pract Res Clin Rheumatol. 2007;21(5):885-906.

6. England BR, Sayles H, Mikuls TR, Johnson DS, Michaud K. Validation of the Rheumatic Disease Comorbidity Index. Arthritis Care Res. 2015;67(6):865-72.

7. Hendrikx J, de Jonge MJ, Fransen J, Kievit W, van Riel PLCM. Systematic review of patient-reported outcome measures (PROMs) for assessing disease activity in rheumatoid arthritis. RMD Open. 2016;2(2):e000202-e.

8. Davergne T, Moe RH, Fautrel B, Gossec L. Development and initial validation of a questionnaire to assess facilitators and barriers to physical activity for patients with rheumatoid arthritis, axial spondyloarthritis and/or psoriatic arthritis. Rheumatology International. 2020;40(12):2085-95.

9. Davergne T, Tekaya R, Sellam J, Tournadre A, Mitrovic S, Ruyssen-Witrand A, et al. Influence of perceived barriers and facilitators for physical activity on physical activity levels in patients with rheumatoid arthritis or spondyloarthritis: a cross-sectional study of 150 patients. BMC musculoskeletal disorders. 2021;22(1):915-.

10. Dupuis F, Cherif A, Batcho C, Massé-Alarie H, Roy JS. The Tampa Scale of Kinesiophobia: A Systematic Review of Its Psychometric Properties in People with Musculoskeletal Pain. The Clinical journal of pain. 2023;39(5):236-47.

11. Government A. Physical activity and sedentary behaviour guidelines – adults (18 to 64 years) – fact sheet. In: Health Do, editor. 2014.

12. Bulley C, Donaghy M, Payne A, Mutrie N. Stages of exercise behaviour change: A pilot study of measurement validity. International journal of health promotion and education. 2008;46(1):4-10.

13. Rosenkranz RR, Duncan MJ, Caperchione CM, Kolt GS, Vandelanotte C, Maeder AJ, et al. Validity of the Stages of Change in Steps instrument (SoC-Step) for achieving the physical activity goal of 10,000 steps per day. BMC Public Health. 2015;15(1):1197-.

**Supplementary Table S2:** Barriers and Enablers to physical activity (PA) reported by participants who met PA guidelines (n = 43) and those who did not (n = 137)

| IFAB questions | Response: | | | p-val^1^ |
| --- | --- | --- | --- | --- |
|  | Barrier | No Effect | Enabler |  |
| **Symptoms** |  |  |  |  |
| Not meeting PA guidelines ^2^ | 84 (61%) | 40 (29%) | 13 (9%) | 0.035 |
| Meeting PA guidelines | 18 (42%) | 22 (51%) | 3 (7%) |  |
| *All* | 102 (57%) | 62 (34%) | 16 (9%) |  |
| **Lack of motivation** |  |  |  |  |
| Not meeting PA guidelines | 70 (51%) | 67 (49%) |  | 0.22 |
| Meeting PA guidelines | 17 (40%) | 26 (60%) |  |  |
| *All* | 87 (48%) | 93 (52%) |  |  |
| **Weather** |  |  |  |  |
| Not meeting PA guidelines | 70 (51%) | 65 (47%) | 2 (1%) | 0.080 |
| Meeting PA guidelines | 14 (33%) | 29 (67%) | 0 (0%) |  |
| *All* | 84 (47%) | 94 (52%) | 2 (1%) |  |
| **A belief that PA will worsen symptoms** |  |  |  |  |
| Not meeting PA guidelines | 35 (26%) | 102 (74%) |  | 0.009 |
| Meeting PA guidelines | 3 (7%) | 40 (93%) |  |  |
| *All* | 38 (21%) | 142 (79%) |  |  |
| **Lack of knowledge re what exercises to do** |  |  |  |  |
| Not meeting PA guidelines | 21 (15%) | 116 (85%) |  | 0.072 |
| Meeting PA guidelines | 2 (5%) | 41 (95%) |  |  |
| *All* | 23 (13%) | 157 (87%) |  |  |
| **Support from friends and family** |  |  |  |  |
| Not meeting PA guidelines | 15 (11%) | 99 (72%) | 23 (17%) | 0.052 |
| Meeting PA guidelines | 0 (0%) | 34 (79%) | 9 (21%) |  |
| *All* | 15 (8%) | 133 (74%) | 32 (18%) |  |
| **Support from health care** |  |  |  |  |
| Not meeting PA guidelines | 10 (7%) | 104 (76%) | 23 (17%) | 0.28 |
| Meeting PA guidelines | 1 (2%) | 38 (88%) | 4 (9%) |  |
| *All* | 11 (6%) | 142 (79%) | 27 (15%) |  |
| **Confidence knowing how to exercise safely** |  |  |  |  |
| Not meeting PA guidelines |  | 95 (69%) | 42 (31%) | 0.018 |
| Meeting PA guidelines |  | 21 (49%) | 22 (51%) |  |
| *All* |  | 116 (64%) | 64 (36%) |  |
| **Knowledge PA good for mood** |  |  |  |  |
| Not meeting PA guidelines |  | 85 (62%) | 52 (38%) | 0.079 |
| Meeting PA guidelines |  | 20 (47%) | 23 (53%) |  |
| *All* |  | 105 (58%) | 75 (42%) |  |
| **Knowledge that PA good for condition** |  |  |  |  |
| Not meeting PA guidelines |  | 72 (53%) | 65 (47%) | 0.084 |
| Meeting PA guidelines |  | 16 (37%) | 27 (63%) |  |
| *All* |  | 88 (49%) | 92 (51%) |  |

PA = physical activity

^1^Chi-Square (exact)

^2^Achieving 600 METs/week and completing two activities a week classified as muscle resistance activity

**Supplementary Table S3:** Source of information regarding physical activity

Survey responses indicating where participants get their information regarding physical activity and where they think they should be getting it

| Information source | Actual source  of information  N=180 | Preferred source of information  N = 177 |
| --- | --- | --- |
| **Rheumatologist** | 43 (23.9%) | 84 (47.5%) |
| **General Practitioner** | 44 (24.4%) | 78 (44.1%) |
| **Physiotherapist** | 48 (26.7%) | 67 (37.9%) |
| **Exercise Physiologist** | 17 (9.4%) | 57 (32.2%) |
| **Other fitness professionals** | 40 (22.2%) | 57 (32.2%) |
| **Friends/family** | 36 (20.0% | 23 (13.0%) |
| **Online** | 44 (24.4%) | 21 (11.9%) |
| **No information** | 37 (20.6%) | 18 (10.2%) |
| **Other^1^** | 17 (9.4%) | 15 (8.5%) |

^1^Examples include occupational therapist, prior PA experience, surgeons, other health professionals. Participants were able to tick more than one option.

**Supplementary Table S4:** Physical activity stage of change assessment

| Stage of change | Total  N=180 (%) | Meeting guidelines N=43 (%) | Not meeting guidelines N=137 (%) | P-val  Fisher’s exact |
| --- | --- | --- | --- | --- |
| **Do you think you do enough physical activity?** |  |  |  | 0.0001 |
| Yes | 73 (41%) | 30 (70%) | 43 (31%) |  |
| No | 90 (50%) | 10 (23%) | 80 (58%) |  |
| Unsure | 17 (9%) | 3 (7%) | 14 (10%) |  |
| **Believes enough physical activity** | Total  N=73 | N=30 | N = 43 |  |
| Have you been taking part in regular physical activity for more than 6 months? |  |  |  |  |
| Yes | 67 (92%) | 30 (100%) | 37 (86%) |  |
| No | 6 (8%) | - | 6 (14%) |  |
| **Does not believe enough physical activity** | Total  N=107 | N = 13 | N = 94 |  |
| Do you intend to start regular PA? |  |  |  |  |
| Yes, in the next month | 56 (52%) | 12 (92%) | 44 (47%) |  |
| Yes, in the next 2-6 months | 30 (28%) | 0 | 30 (32%) |  |
| No | 21 (20%) | 1 (8%) | 20 (21%) |  |
